# Dietary Habits Bursting into the Complex Pathogenesis of Autoimmune Diseases: The Emerging Role of Salt from Experimental and Clinical Studies

**DOI:** 10.3390/nu11051013

**Published:** 2019-05-05

**Authors:** Rossana Scrivo, Carlo Perricone, Alessio Altobelli, Chiara Castellani, Lorenzo Tinti, Fabrizio Conti, Guido Valesini

**Affiliations:** Department of Internal Medicine and Medical Specialties, Rheumatology, Sapienza University of Rome, Rome 00185, Italy; rossana.scrivo@uniroma1.it (R.S.); alessioaltobelli@gmail.com (A.A.); castel.chia@gmail.com (C.C.); lorenzo.tinti.cv@gmail.com (L.T.); fabrizio.conti@uniroma1.it (F.C.); guido.valesini@uniroma1.it (G.V.)

**Keywords:** sodium chloride, salt, environmental factors, autoimmunity, rheumatoid arthritis, systemic lupus erythematosus, multiple sclerosis, inflammatory bowel disease, Th17 cells, Treg cells

## Abstract

The incidence and prevalence of autoimmune diseases have increased in Western countries over the last years. The pathogenesis of these disorders is multifactorial, with a combination of genetic and environmental factors involved. Since the epidemiological changes cannot be related to genetic background, which did not change significantly in that time, the role of environmental factors has been reconsidered. Among these, dietary habits, and especially an excessive salt, typical of processed foods, has been implicated in the development of autoimmune diseases. In this review, we summarize current evidence, deriving both from experimental models and clinical studies, on the capability of excessive salt intake to exacerbate proinflammatory responses affecting the pathogenesis of immune-mediated diseases. Data on several diseases are presented, including rheumatoid arthritis, systemic lupus erythematosus, multiple sclerosis, and Crohn’s disease, with many of them supporting a proinflammatory effect of salt. Likewise, a hypertonic microenvironment showed similar effects in experimental models both in vivo and in vitro. However, murine models of spontaneous autoimmune polyneuropathy exposed to high salt diet suggest opposite outcomes. These results dictate the need to further analyse the role of cooking salt in the treatment and prevention of autoimmune diseases, trying to shape a fine tuning between the possible advantages of a restricted salt intake and the changes in circulating metabolites, mediators, and hormones which come along salt consumption and could in turn influence autoimmunity.

## 1. Introduction 

Over time, the spectrum of autoimmune disorders has constantly increased, paralleling remarkable advances in understanding the phenotype and functioning of the immune cells, the mechanisms sustaining the interplay of the immune responses, and the epigenetic deregulations in genetically susceptible hosts [1]. In addition, environmental triggers, including infections, hormones, drugs, cigarette smoking, pollutants, and dietary habits are deemed responsible for the increased incidence and prevalence of autoimmune diseases observed in the last years in Western countries, not completely excused from genetic background, better diagnosis, and improved access to healthcare services [2,3]. Among environmental factors, dietary habits have been unequivocally associated to severe conditions, such as hypertension, heart disease, stroke, and cancer [4,5,6,7]. More recently, vitamin D deficiency was implicated in the development of immune-mediated diseases [8,9,10], and an excessive salt (sodium chloride, NaCl) intake may also have similar effects. Sodium is the most abundant extracellular cation in humans, where it is critical in maintaining extracellular fluid volume and osmotic balance and in generating the membrane potential of cells [11]. Most dietary sodium is consumed as common salt, i.e., sodium chloride. The World Health Organization (WHO) recommends limiting the salt intake to less than 5 g per day [12] in order to reduce the risk of developing hypertension [13], cardiovascular [14] and kidney diseases [15]. Even though in vitro and murine studies have shown that high sodium intake is associated to a better response to pathogenic micro-organisms [16], on the other hand, novel original studies have shown that an excessive salt intake may promote the development of immune-mediated diseases in mice models, such as collagen-induced arthritis, experimental autoimmune encephalomyelitis, and experimental colitis [17,18,19]. Moreover, it was demonstrated that excessive salt intake favors pro-inflammatory responses in patients with rheumatoid arthritis (RA), systemic lupus erythematosus (SLE), multiple sclerosis (MS), inflammatory bowel disease (IBD) [20,21,22,23,24,25,26,27,28,29,30]. These observations carry practical implications, since in Western countries salt is often added to food and consumed beyond the recommended amounts [12]. Therefore, excessive salt consumption may be considered a modifiable risk factor in the frame of the complex pathogenesis of autoimmune diseases.

Here, we summarize current evidence, deriving both from experimental models and clinical studies, on the capability of excessive salt intake to exacerbate proinflammatory responses affecting the pathogenesis of immune-mediated diseases.

## 2. Salt and Murine Models 

The role of dietary factors on immune functions has been explored in several murine models. Recently, two independent studies showed that an excess salt intake can modulate T cells immune response in one to three weeks. Specifically, it was shown that high concentrations of NaCl promote the differentiation of T helper (Th) lymphocytes toward the proinflammatory Th17-driven immune response via the serum- and glucocorticoid-induced kinase 1 (SGK1) mediator [31,32]. SGK1 is one of the major kinases that regulates Na^+^ intake by phosphorylation of epithelial sodium channels [31]. SGK1 has also an important role in controlling the balance between regulatory T (Treg) and Th17 cells. In fact, it activates Forkhead box protein O1 (FOXO1), a regulator of the expression of Foxp3 (a key transcription factor in Tregs), whose loss favors the development of autoimmunity [31,33]. In addition, FOXO1 decreases the expression of interleukin-23 receptor (IL-23R) through directly binding to the IL-23R promoter and inhibiting RORγt-mediated IL-23R transactivation. 

The phosphorylation deactivates FOXO1 and promotes uncontrolled transcription of RORγt-mediated IL-23R. Indeed, the loss of SGK1 makes mice highly resistant to the development of autoimmunity, and SGK1 deficiency results in the inhibition of Th17 development and in the enhancement of Treg activity [34]. It was shown that naïve T cells activated in the presence of additional NaCl significantly upregulate SGK1, IL-23R, and other genes associated with Th17 development, while a sodium-induced increase in Th17 development and IL-23R expression was not observed in SGK1-deficient T cells [31]. In in vivo studies, wild type (WT) and SGK1-deficient mice were fed with a high-salt diet (HSD), with the WT group showing a marked increase in the frequency of Th17 cells in the gut lamina propria (LP). At the same time, SGK1-deficient mice showed a lower enhancement of Th17 cell frequency in the LP. No increase in IFN-γ production in any of the mice fed with HSD was documented [31]. These data lead to hypothesize that an increased sodium concentration may induce the Th17 cell phenotype through SGK1 [31]. In fact, NaCl, lauric acid (LA) and, to greater extent, a combination of both were shown to stimulate the differentiation of Th17 cells, decrease Th2 differentiation and magnify the production of IL-17 in the Th17 differentiating culture. The expression of SGK1 was mainly induced by NaCl or by the combination of NaCl and LA. Furthermore, NaCl alone and NaCl together with LA increased in the same extent Th1 and Th17 cell frequencies in the central nervous system (CNS) [35]. The same SGK1 is responsible for Na+ transport and salt homeostasis in other cells [36]. Even in unimmunized mice, enhancement of Th17 differentiation was observed in vivo in the gut and gut-associated lymphoid tissue through modest increase of the NaCl concentration and consequent induction of SGK1 expression [31]. 

Krementsov et al. evaluated WT C57BL6/J and SJL/JCrHsd mice, which were given either a low-sodium or a high-sodium diet [37]. The authors showed a dichotomic behavior of experimental autoimmune encephalomyelitis (EAE) in response to dietary sodium in males and females, suggesting genetic control. Among the candidate genes, Slc9a3r2 (solute carrier family 9 [sodium/hydrogen exchanger], member 3 regulator 2), located within the chromosome 17 locus, was differentially expressed between the sodium-responsive (B6 and SJL) and the sodium-unresponsive strains (129Sv). Moreover, the authors showed that high dietary sodium causes elevated serum sodium and increases blood-brain barrier permeability, thus leading to a more severe brain pathology. Of note, at least in this experiment, there was not a change in encephalitogenic T cell responses, neither in the enhancement of Th1 or Th17 response. In addition, Treg cells generation seemed not to be influenced by dietary sodium [37].

The complexity of the issue is even more evident when considering the experimental study by Huehnchen et al. in murine models of spontaneous autoimmune polyneuropathy, where a high salt diet was associated with a decreased expression of IL-17, an improved demyelination and a reduced infiltration of the sciatic nerve [38]. The authors hypothesized that the NOD background could have had an influence on the gut microbiota specific for each mouse strain. Indeed, as shown by Wilck et al., it is known that high salt provokes profound changes in gut microbiota composition. The authors found a decrease in several species including the genera Lactobacillus, Oscillibacter, Pseudoflavonifractor, Clostridium XIVa, Johnsonella, and Rothia, while others increased under salt stimulus, such as Parasutterella spp. These changes may be pathogenetic, since L. murinus seems to ameliorate active EAE and reduce salt-sensitive hypertension through production of indole-3-lactic acid, which in turn reduces Th17 polarization in a dose dependent manner [39].

In another study, a further detrimental mechanism was suggested for a high salt diet influencing the development of autoimmunity. In particular, in the context of a murine SLE model, it was shown that NaCl increases the frequency of T follicular helper cells (Tfh) cells in peripheral blood mononuclear cells (PBMCs) and CD4+T cells and promotes Tfh cell differentiation. Intriguingly, a high salt diet seems to accelerate the progression of lupus in MRL/lpr mice but not MRL/mpj and Balb/c mice, and, finally, NaCl seems to induce DNA hypomethylation of CD4+T cells and to enhance the expression of the hydroxyltransferases TET2 and TET3. It is possible that sodium chloride enhances the expression of map3k1 and SPN and induces DNA hypomethylation of genes that are involved in the immune response pathway such as inducible T cell costimulator (ICOS), programmed cell death protein 1 (PDCD1), signal transducer and activator of transcription 1 (STAT1), and SH2 domain-containing protein 1A (Sh2d1a). Thus, epigenetic is another mechanism by which salt modulates enhanced autoimmunity [40]. 

To conclude on the studies on murine models, the question remains: Which are the most affected cells? It is most likely that the murine model, thus the genetic background of the mouse, is the single most important variable influencing the immune response to salt. Nonetheless, Vaartjes et al. showed that increased salt exposure affects CD4+ T-cells towards a proinflammatory phenotype characterized by increased secretion of IFNγ and IL-17A. Moreover, macrophages show increased production of both TNFα and IL-10. DSS-induced colitis seemed to be the most significantly affected model by moderate salt intake [41]. In this interesting study, the authors were able to confirm the effects of salt on autoimmunity. They suggested that “reasonably increased amount of salt,” meaning 1% NaCl, affects both lymphoid and myeloid cells ex vivo, but, at least in vivo, the effects seem less pronounced in terms of CD4+ T-cell responses compared with macrophage-dependent pathologies.

## 3. Salt and Innate and Adaptive Immune Response 

Several evidences confirm the role of salt in the regulation of both innate and adaptive immunity. Studies performed using 3 Tesla ^23^Na^+^ magnetic resonance imaging (MRI) and high magnetic strength (7 Tesla) MRI showed that sodium can accumulate in the human skin producing a hypertonic environment [42,43]. This was observed in inflamed tissues [44] as well as in the lymphatic organs [45]. Monocyte phagocytic system is involved in the regulation of sodium storage in the skin in response to the local hypertonic environment. Also, macrophages can change their function in response to osmotic stress [46]. Interestingly, in vitro and murine studies have shown that high sodium intake is associated to a better response to pathogenic micro-organisms such as Escherichia coli and Leishmania major [16]. This phenomenon would represent the expression of an ancient defense mechanism against pathogens, confirming the ability of sodium to modify the immune response. 

### 3.1. Innate Immunity 

The effects of increased sodium intake on innate immunity were mainly investigated on the monocyte/macrophage series. Most studies were conducted in vitro or in murine models, with only a few in humans. 

In vitro studies were typically carried out by adding 40 mM sodium to cell cultures, reaching a sodium concentration of 179 mM, based upon results in mouse models [47]. Moreover, adding 80 mM urea, 20 mM MgCl_2_ or 80 mM mannitol, an equivalent osmolality and tonicity of 40 mM NaCl was obtained, which is crucial to rule out the effect of hyperosmolarity on changes in the immune response [18,48,49]. Macrophages are able to migrate to excess NaCl. The maximum migration capacity is obtained when the difference in sodium concentration is 40 mM and equals 60% as compared to cells stimulated with the CXCL12 chemokine [50]. Furthermore, salt may induce the production of INF-β in human and murine macrophages through the p38 MAPK/ATF2/AP1 signaling pathway, enhancing the immune response sensing and signaling when challenged with viruses [51].

Macrophages are able to develop 2 different types of immune response. The M1 phenotype (stimulated by TLR ligands and IFN-γ) is characterized by the expression of high levels of proinflammatory cytokines promoting the Th1 and Th17 responses. In contrast, M2 phenotype exhibits immunoregulatory functions and is involved in tissue repair mechanisms [52]. However, the activation spectrum of macrophages is presently better defined by means of the activator (i.e., lipopolysaccharide or IL-4), rather than by the M1/M2 phenotype [53]. In this regard, an excessive salt intake drives a proinflammatory activation state, termed M(Na), in both human and mouse macrophages. In vitro, M(Na) is characterized by the expression of genes coding for several proinflammatory mediators, such as CCL2, CCL8, CXCL1, CXCL2, IL-1β, IL-8, CCR2, TLR3, TLR4, and CD14. In contrast, the expression of genes coding for anti-inflammatory mediators, including CCL18, CCL22, TREM2, MRC1, is suppressed. Moreover, excessive salt may potentiate M(LPS) and suppress M(IL-4), that configure the classical pro- and anti-inflammatory macrophage activation state, respectively. The induction of the proinflammatory properties of M(Na) by salt is mediated by the p38/cFos/AP1 and Erk1/2/cFos/AP1 pathways. The former also enhances M(LPS) by salt, whereas Erk1/2/STAT6 pathway mediates the suppression of the anti-inflammatory response [51]. Also, high salt alters in vitro the activation of bone marrow-derived mouse macrophages stimulated by IL-4 and IL-13, reducing the anti-inflammatory activation state and their ability to suppress effector T cell proliferation. This seems to be secondary to the inhibition of the increased glycolysis and oxidative phosphorylation necessary for M(IL-4/IL-13) activation. The alteration of AKT/mTOR signaling, known to be important for nutrient sensing and orchestrating the metabolic “switches” for immune cell activation, is involved in the reduction of anti-inflammatory macrophage activation [50]. 

In humans, a sodium-rich diet increased the levels of circulating monocytes in a small cohort of six healthy male volunteers. They were exposed to dietary salt reduction stepwise from 12 to 9–6 g salt per day. Each salt level was maintained on average for 50 +/− 10 days. The salt reduction phase was followed by a 30-day period of 12 g intake. The absolute number and percentage of peripheral monocytes were significantly higher at each 12 g salt stage compared with lower salt levels. Moreover, the macrophages from the volunteers subjected to ex vivo mitogen stimulation assay produced a smaller amount of proinflammatory cytokines (IL-6 and IL-23), while increasing the anti-inflammatory cytokine IL-10 throughout the reduction of salt intake [54].

In addition to macrophages, salt affects dendritic cells. In a high sodium microenvironment, splenic dendritic cells from mice are activated and produce IL-1β. Activation of dendritic cells is mediated by intake of sodium through amiloride-inhabitable sodium channels. This leads to intracellular calcium influx, via the sodium-calcium exchanger, and activation of protein kinase C (PKC). Activated PKC phosphorylates p47 protein that induces the assembly of the NADPH oxidase enzyme, the increased formation of superoxide production, and the immunogenic isolevuglandins (highly reactive γ-ketoaldehydes) in dendritic cells. Moreover, high salt-activated dendritic cells increase the expression of the B7 ligand CD86. These cells are able to stimulate the production of IL-17A and IFN-γ by T lymphocytes [55].

All these data lead to hypothesize that an excessive and chronic intake of high levels of salt promotes a proinflammatory state of the innate immune system that could either be an effective mechanism of defense against pathogens or participate in the development of autoimmune diseases.

### 3.2. Adaptive Immunity 

There is evidence that salt may disrupt the activity of murine and human Treg lymphocytes both in vivo and in vitro. Indeed, a co-culture of Treg lymphocytes and CD4+ naïve effector cells was used to demonstrate the suppression of inflammation by adding 40 mM NaCl. An impairment of the suppressive proficiency of Tregs was shown. Furthermore, purified and activated Tregs exposed to high salt concentration switched to the Th1 pattern, producing many proinflammatory mediators, such as CXCR3, IFN-γ, and TBX21. Also, Th17-related mRNA expression increased, including RORC (RAR-related orphan receptor C), CCR6, IL-17A, and IL-17F [56]. 

The proinflammatory activity of sodium chloride has been also demonstrated in mice with graft versus host disease (GvHD). One group of mice was fed with a normal salt diet, the other with HSD: Interestingly, in the former group, human Tregs were able to stop GvHD; in the latter, they were not and, moreover, they produced higher quantities of IFN-γ [56].

As far as rheumatic diseases are concerned, T cells isolated from patients with RA and osteoarthritis (OA) showed that NaCl increase Th17 differentiation in a dose-dependent manner. Using culture media and flow cytometry, 40 mM NaCl was best successful in increasing the expression of RORγt and IL-17, while T cells showed apoptosis at a concentration of 60 mM NaCl. IL-17 was also searched in synovial fluid (SF) together with the concentration of Na+: a higher concentration of such ion was documented in SF from patients with RA with respect to OA patients. Interestingly, Na+ concentration in RA SF was the same registered in the culture media exposed to 40 mM NaCl (104.4 mM). Synovial Na+ concentration above 160 mEq/L (corresponding to the Na+ concentration when 60 mM were added) seemed not to favor lymphocyte proliferation, suggesting that an hypernatremic SF, without exceeding in Na+ concentration, may favor inflammation and Th17 cell proliferation [17].

Moreover, a subset of Tregs, known as iTregs (TGF-β-induced CD4+Foxp3+ regulatory T cells), showed increased proliferation in the presence of high salt concentrations. However, polarisation towards Th1 or Th17 was not observed, as confirmed by a low production of IFN-γ and IL-17 in vitro. Therefore, given the stability in high-salt milieu, it was hypothesized that a manipulation of iTreg might be investigated in the treatment of autoimmune diseases [57].

To sum up, many studies suggest that salt influences the suppression of Treg proliferation (but not of iTregs), promotes the switch of Tregs versus Th1 lymphocytes, and can induce the Th17 response (Table 1).

## 4. Clinical Studies 

Clinical studies investigating the role of dietary salt intake on the progression and manifestation of chronic immune-mediated diseases are available for RA, SLE, MS, and Crohn’s disease (Table 2). However, evidence also exists in healthy volunteers investigated in a pilot study [39]. Eight participants received an increased salt intake for 14 days using slow-release NaCl tablets in addition to their accustomed diets. Analysis of peripheral blood lymphocytes using flow cytometry revealed a significant increase in CD4+IL-17A+TNF+ Th17 cells after HSD with respect to baseline. Furthermore, the effect of HSD on the human gut microbiome were explored, showing a loss of Lactobacilli compared to non-Lactobacillus species in fecal samples after high salt challenge [39].

When considering pathologies, most studies were carried out in patients with RA, with the aim to investigate whether the intake of sodium chloride might favor the disease development [20,21,22] or severity [23].

In particular, a Swedish nested case-control study used prospective data from a screening and intervention program for risk factors of cardiovascular diseases, the Västerbotten Intervention Programme (VIP). This study included 386 RA patients and 1886 matched controls, who were asked to fill in a food questionnaire to quantify the amount of sodium intake a median of 7.7 years before the onset of symptoms of RA (or corresponding date in the controls). Overall, salt intake was neither associated with the presence of antibodies to citrullinated peptides (ACPA) nor with the development of RA. However, smokers in the highest tertile of sodium consumption showed more than a two-fold increase in risk of developing RA with respect to the subjects in the lowest tertile (OR 2.26; 95% CI 1.06–4.81) [20]. These results were not replicated in a Spanish, cross-sectional study based on the SUN (‘‘Seguimiento Universidad de Navarra’’) cohort, a dynamic prospective investigation launched in 1999 by the Department of Preventive Medicine and Public Health of the University of Navarra. The main objective of the SUN cohort is to study the influence of diet, habits, and physical activity on diseases through follow-up questionnaires exploring diet, lifestyle, risk factors, and medical conditions. The nested analysis aimed at investigating a possible association between sodium intake, estimated through a validated questionnaire, and the development of RA. Among 18,555 subjects, 392 self-reported a diagnosis of RA. The mean daily sodium intake was 3.47 g. Individuals in the highest quartile (>4.55 g/day) were at higher risk of developing RA (OR 1.4; 95% CI, 1.1–1.9; *p* = 0.02), even after adjustment for confounding factors, but never smokers with a high sodium intake had a significantly higher risk compared to ever smokers (*p* = 0.007). Dose-dependent association was replicated in the case-control study used for sensitivity analyses [21]. Despite the different conclusions, both the Swedish and the Spanish study suggest that salt intake, assessed by the French validated food frequency questionnaire (FFQ) [58], may be among the environmental factors involved in the development of RA. Following these results, another Swedish study took advantage from data obtained in the EIRA (Epidemiological Investigation of Rheumatoid Arthritis) project during 2009–2011. The objective was to investigate whether sodium consumption, evaluated by means of a self-reported questionnaire, could affect the development of ACPA-positivity among smokers and also to evaluate the effect of smoking on ACPA-positivity according to the amount of sodium consumption [22]. Interestingly, smoking status was found to be associated with ACPA-positive RA only in patients with medium or high sodium intake (OR 1.7 and 2.09 for ever smokers and heavy smokers, respectively). Likewise, medium or high sodium consumption was associated with an increased risk of ACPA-positive RA among smokers (OR 1.3 and 2.1 in ever smokers and heavy smokers, respectively). However, no interaction between salt and SGK1 polymorphisms, in relation to ACPA-positivity, was demonstrated. Hence, despite the low number of patients in the heavy smoking/low sodium stratum and difficulties to accurately measure sodium intake via questionnaires, this study adds further evidence about the impact of high salt intake on immune reactions typical of RA, especially in the presence of other environmental factors such as smoking [22]. Sodium intake was also found to be associated with RA severity in 24 patients with early RA and 24 controls matched by age, gender, and body mass index participating in a case-control study. Here, sodium intake was evaluated by 24-hour urinary sodium excretion, which is considered the most reliable method for estimating sodium intake [59,60], and resulted to be increased in RA patients than matched controls (2.849 ± 1.350 vs 2.182 ± 751,7 mg/day, *p* = 0.039), even after adjustment for the use of hypertensive drugs, nonsteroidal anti-inflammatory drugs, and smoking, and was also greater in those with radiographic erosions (median [IQR] 4.232 (2.898–5.083) vs 2.231 (1.737–2.979) mg/d sodium, *p* = 0.028). Twenty-four-hour urinary sodium excretion was neither affected by disease activity evaluated by DAS28-CRP, nor by autoantibody status [23]. 

Differently from these studies, we assessed the biological effects of a low salt diet intervention in patients with RA or SLE. Given the harmful effects of excess sodium consumption, patients underwent a dietary regimen starting with a restricted daily sodium intake followed by a normal-sodium daily intake, thus avoiding the exposure to a surplus in sodium dietary intake. The study was planned to cover a five-week period: in the first three weeks patients were asked to maintain a low-sodium dietary regimen following the indications in the leaflet given to each of them. After that, patients entered the last two weeks of the study, where they followed a normal-sodium dietary regimen, meaning that they were supposed to meet the WHO recommendations requiring not to exceed 5 g salt/day. The aim of the study was to recognize possible changes in Th17 and Treg lymphocyte number in relation to the two different dietary conditions. Adherence to the dietary regimen was deemed as an unavoidable requirement in order to provide believable research findings. Therefore, we used 24-hour urinary sodium excretion. Fourteen patients with RA and 15 with SLE complying with the dietary regimen were identified. All the patients showed a reduction in the frequency of Th17 cells after the first three weeks of low-sodium dietary regimen, and an increase after the two further weeks characterized by an augmented salt intake, albeit within the limits of WHO recommendations. Conversely, Treg lymphocytes exhibited the opposite trend: an increase was observed in the first three weeks, followed by a reduction over last two weeks [24]. Another study, characterized by a cross-sectional, observational design, reached different results when exploring the association between salt intake and immune parameters in the peripheral blood from patients with RA and SLE and healthy subjects. Salt intake was estimated through both 24-hour urinary sodium excretion and a written questionnaire. Accordingly, the subjects enrolled were classified as having low-salt intake (LSI; less than 5 g/day of NaCl) and high-salt intake (HIS; 5 or more g/day of NaCl). Similar levels of Foxp3+ and CD69+ Treg cells as well as of Th17 cells were observed in the peripheral blood from LSI and HSI patients or controls. However, a positive correlation was detected between sodium intake and levels of Foxp3+ Treg cells in SLE and a negative association between CD69+ Treg cells and sodium intake in RA. The suppressor activity of Foxp3+ and CD69+ Treg cells was similar in LSI and HSI patients or controls [25]. The apparent contradiction between these results and previous reports might be explained by several factors, including the genetic background of the population studied (Mexican mestizo), the diet, the degree of sun exposure and vitamin D levels, and methodological differences.

Some controversies also exist in studies performed in patients with MS. In an observational study, 70 adult patients with relapsing-remitting MS were enrolled to investigate the relationship between salt intake, evaluated by sodium excretion in urine samples, and clinical and radiological disease activity over a two-year follow-up. The clinical evaluation was obtained both by the expanded disability status scale-EDSS performed at baseline and at the end of follow-up and the number of relapses during follow-up. The radiological activity was assessed by brain and spinal cord MRI at baseline and at three- or six-month follow-up visits on a 1.5-T MRI unit. A positive correlation between exacerbation rates and sodium intake was found in a multivariate model adjusted for age, gender, disease duration, smoking status, vitamin D levels, body mass index and treatment. The exacerbation rate was 2.75-fold (95% CI 1.3 to 5.8) and 3.95-fold (95% CI 1.4 to 11.2) higher in patients with medium (between 2 and 4.8 g/day) or high (more than 4.8 g) sodium intake, respectively, compared with the low-intake (below 2 g/day) group. Additionally, individuals with high-sodium intake had a 3.4-fold greater chance of developing a new lesion on the MRI and on average had eight more T2 lesions on MRI. A similar relationship was found in cross-sectional analysis of the independent replication group of 52 patients with relapsing-remitting MS [26]. Conversely, sodium intake was irrelevant in the BENEFIT study, a randomized clinical trial designed to assess whether a high-salt diet, as measured by 24-hour urine sodium levels, is associated with faster conversion from clinically isolated syndrome to MS and disease activity and disability [27]. In the same way, two studies performed in pediatric patients did not show any association between sodium intake and MS [28] or relapse of neurological disease [29]. Finally, only one study examined the relationship between salt consumption and Crohn’s disease (CD). Information on lifestyle and diet of women from the Nurse Health Studies were analyzed, producing no evidence of a possible association between dietary salt intake and incident cases of CD [30].

## 5. Conclusions 

The complex pathogenesis of immune-mediated disorders, involving the interaction of genetic, hormonal, immunological, and environmental factors, has inspired the concept of “mosaic of autoimmunity” to underline the heterogeneous substructure generating a variety of clinical manifestations likewise. Curiously, the incidence and prevalence of autoimmune diseases have increased in Western countries over the last years. Since the epidemiological changes cannot be related to genetic background, which did not change significantly in that time, the role of environmental factors has been reconsidered. Among these, dietary habits have captured much interest in autoimmune diseases, following evidence that they may affect the development of cardiovascular diseases and cancer. Indeed, several studies investigated the role of sodium chloride in the occurrence of autoimmune diseases, albeit no definite conclusion can be drawn. Experimental models and clinical studies in RA, SLE, MS, and IBD concur to sustain the assumption that excessive salt, typical of processed foods, may drive the activity of immune cells towards a proinflammatory response. However, some murine models and clinical studies of autoimmune diseases suggest opposite outcomes. These results dictate the need to further analyze the role of cooking salt in the treatment and prevention of autoimmune diseases, trying to shape a fine-tuning between the possible advantages of a restricted salt intake and the changes in circulating metabolites, mediators, and hormones, which are related to salt consumption and could in turn influence autoimmunity.

## Figures and Tables

**Table 1:** Effect of sodium on immune response in vitro and in vivo.

Immune Response	Effect	Pathway	References
**Innate immunity**			
Macrophages	Activation of migration	-	[50]
	Production of INF-β	p38 MAPK/ATF2/AP1	[51]
	Induction of M(Na) proinflammatory phenotype	p38/cFos/AP1 and Erk1/2/cFos/AP1	[51]
	Strengthening of proinflammatory M1 response	p38/cFos/AP1 and Erk1/2/cFos/AP1	[51]
	Suppression of anti-inflammatory M2 response	Erk1/2/STAT6 - AKT/mTOR signaling	[48,51]
Dendritic cells	Production of IL-1β	PKC/p47/ NADPH oxidase enzyme	[55]
	Production of IL-17A and IFN-γ by T lymphocytes	-	[55]
**Adaptive immunity**			
Lymphocytes	Suppression of Treg proliferation (but not iTreg)	-	[56,57]
	Switch of Treg versus Th1	-	[56]
	Induction of Th17 response	SGK1	[31]

M(Na), Na^+^ induced macrophages; iTreg, TGF-β-induced CD4+Foxp3+ regulatory T cells; SGK1, serum-and glucocorticoid-induced kinase 1; MAPK, mitogen-activated protein kinase; ATF2, activating transcription factor 2; cFos, transforming member of the Fos protein family; AP1, activator protein-1; Erk1/2, extracellular signal-regulated kinase1/2; STAT6, signal transducer and activator of transcription 6; PKC, protein kinase C; -, unknown.

**Table 2:** Effect of sodium intake on immune response in published studies.

Disease	Evidence	Design of the Study	References
Rheumatoid arthritis	Salt as one of the environmental factors involved in the development of RA (conflicting data) Salt modules Th17/Treg ratio in RA patients	Case-control	[20,22]
		Cross-sectional case-control	[21,25]
		Clinical trial	[24]
Systemic lupus erythematosus	↓ Th17 and ↑ Treg with low salt intake	Clinical trial	[24]
Multiple sclerosis	Conflicting data	Prospective case-control	[26]
		Nested case-control from a randomized clinical trial	[27]
		Cross-sectional case-control	[28,29]
Crohn’s disease	No evidence of association between dietary salt intake and CD	Nested case-control from a prospective study	[30]

RA, rheumatoid arthritis; CD, Crohn’s disease. ↓ reduction; ↑ increase.
